# Supplementary material for: Disentangling the mutational effects on protein stability and interaction of human MLH1
Source: PLoS Genet. 2025 Apr 28;21(4):e1011681. doi: 10.1371/journal.pgen.1011681 (PMC12064032; doi:10.1371/journal.pgen.1011681)
Supplement: Attachment — Submitted filename: MLH1 Response to Reviewers.pdf [file pgen.1011681.s020.pdf]

Our point-by-point response to the raised issues

Reviewer #1 The manuscript "Disentangling the mutational effects on protein stability and interaction of human MLH1" by Larsen-Ledet and Stein reports on a systematic experimental assessment of the impact of MLH1 variants on protein abundance/stability and intraction with its partner PMS2. The study seems well-conducted and the results are presentedin a clear and critical manner. The data themselves are an important contribution to the field and the authors highlight some interesting findings with respect to the evolutionary and functional properties of the protein. I think some aspects of the computational analysis would benefit from a deeper look (see below).

Our response:
We thank the reviewer for the kind comments and for taking time to evaluate our work and provide valuable feedback.

1) The authors found that substitutions of surface-exposed residues in the central α-helix spanning position 605-627 exhibited slightly decreased interaction with PMS2, without affecting the MLH1 abundance. They speculated that the central α-helix spanning position 605-627 could be a yet unknown binding site for PMS2, and tested this hypothesis by running AF on MLH1 and a fragment of PMS2 (supp fig. 10). What is the Predicted Aligned Error (PAE) corresponding to that interface? Is it higher than the interface obtained with the full-length PMS2? More generally, PAE is a better metric than pLDDT to assess the quality of complex interface, and hence it would be desirable to report the PAE matrices for the predicted 3D models. The interface predicted TM-score (iPTM) is also now included in ColabFold, the authors may consider reporting this metric as well. It would also be interesting to report the differences between the unbound protein structures and the bound conformations in the predicted 3D model.

Our response:
We thank the reviewer for nudging us to more thoroughly investigate this putative interaction interface. Upon closer inspection, we realized that the 605-627 helix docked in isolation is oriented with its N-terminal residue closest towards the beta-strand-interface. In combination with the linker length, this makes it a very unlikely binding site to be adopted during interaction of the full-length proteins, though it is possible that the 605-627 helix participates e.g. in an encounter complex.
We have subsequently modelled the interaction of both full-length proteins, and identified another helix further N-terminal in the linker (residues 409-431) that is predicted to bind to the region where our yeast-two-hybrid experiments have shown changes in interaction upon mutation. This is illustrated in the new Supplementary Figure S10 and discussed on page 11 in the revised manuscript.
In order to get comparable iPTMs, we regenerated the structure of the interaction of the C-terminal domains with AlphaFold 3. The RMSD to the AlphaFold Multimer model is 0.85Å, which we consider low enough to not update the figures in the manuscript where the model is

used for visualization purposes. The ipTM of the interaction of the C-terminal domains is 0.86 while the ipTM of the full-length complex is 0.48. This is considerably lower. We also generated an intermediate construct that has both C-terminal domains and enough of the PMS2 linker to include both helices, and the ipTM score of that is 0.84, very similar to our original C-terminal model. We thus suspect that the lower score of the full-length complex is due to the interface between the N-terminal domains and/or the many disordered elements in both linker regions. Our experimental screens did not contain mutations in the N-terminal domain.

2) "Interestingly, at position 540, the substitution to glutamic acid had no effect on the interaction with PMS2, whereas the substitution to aspartic acid displayed complete loss-of-interaction". Could the authors rationalise this observation by analysing the network of interactions at the interface?

Our response:
Indeed, it is puzzling that we observe such diverse effects at position 540 for substitutions of aspartic acid vs. glutamic acid and arginine vs. lysine. We generated the four variant structures using Rosetta and calculated the ΔΔG of binding using Rosetta's InterfaceAnalyzer for aspartic acid (ΔΔG = 1.80), glutamic acid (ΔΔG = 2.16), arginine (ΔΔG = 5.68), and lysine (ΔΔG = 5.06). These values do not correlate well with our experimental interaction scores. Hence for the moment, we assume that these experimental measurements are beyond the predictive capabilities of the tools we tested.

3) Since the authors have produced experimental data for abundance and hence stability, they could assess the accuracy of Rosetta score with respect to this phenotype replace Rosetta score by the experimental abundance scores in Fig 4B; this would likely extend the main cloud of points and improve interpretation of the results. Are the variants impacting interaction (which would be strongly red or strongly blue) but not stability (they would be on the left) under selective pressure (at the bottom)? Right now, it's a bit difficult to draw conclusions because there are some points with Rosetta scores close to zero (left) and with very negative GEMME scores (bottom) that are red on panel A (indicating strong impact on stability).

Our response:
We thank the reviewer for suggesting this comparison. We now include a scatter plot where we replace the Rosetta scores with our experimental abundance scores (new **Supplementary Fig. 12**) and reference it in the revised manuscript (line 278). Indeed, as pointed out by the reviewer, most loss-of-interaction variants have very negative GEMME scores and have reduced abundance levels.

4) What are the residues with very negative GEMME scores that do not show up in any of the experimental assays. What are their positions in the protein sequence? Do they cluster in 3D so as to form some kind of interface?

Our response:
These variants are found in position G532 and Q542, which are located in the first and third β-strand of the canonical interface, respectively, and points outwards towards PMS2. Despite being predicted to be important for MLH1 function (very negative GEMME scores), they are predicted to have little impact on the stability of the protein (neutral Rosetta scores), which is typically found for residues that are important for function but not stability (see e.g. Cagiada *et al., Nat Comms* 2023).
Our experimental data shows that substitutions to mainly hydrophobic and aromatic residues at these positions have no impact on the abundance levels nor the interaction with PMS2. In addition, we confirm in low-throughput that Q542L (which is one of the variants in question) has wild-type-like abundance levels and interaction with PMS2. Therefore, the most likely interpretation of the negative GEMME scores is that these variants are important for other properties related to MLH1 function (e.g., interaction with other partners like Exo1). Given that GEMME operates on MSAs, it could also be that these residues are important for the interaction in other species, but not for the human proteins we are testing here. Lastly, we cannot rule out that there is a subtle effect on the interaction with PMS2, outside the dynamic range of our experiments but measurable on the evolutionary scale that affects the input to GEMME.

Reviewer #2 This manuscript by Larsen-Ledet and Stein investigates the effects of missense mutations on the stability and protein-protein interaction (PPI) of the human MLH1 protein, which is implicated in Lynch syndrome. The authors use two high-throughput yeast-based assays to systematically examine the effects of 4839 (94%) MLH1 missense and nonsense variants in the C-terminal domain on protein abundance (DHFR-PCA) and interaction (Y2H) with its obligate binding partner, PMS2. One of the study's key finding is that the majority of MLH1 variants that lose interaction with PMS2 also reduce MLH1 stability, suggesting that the loss of interaction might be difficult to separate from loss of stability and reduced cellular abundance. This suggests a strong link between protein stability and interaction for MLH1 and the authors propose that this could be a generally feature of interactions that are driven by large and well structured regions. The authors propose a novel distal region in the C-terminal domain of MLH1 where substitutions can cause both decreased and increased binding with PMS2, independent of changes in abundance. This region is proposed to be a novel interaction site. The authors also compare their experimental data with computational predictions of thermodynamic stability (using Rosetta) and evolutionary conservation (using GEMME) and find a good qualitative agreement between the predictions and experiments but do not really quantify these findings. Lastly, the authors analyse the clinical relevance of their findings by comparing the experimental data to variant classifications in the ClinVar database and population allele frequencies from gnomAD. They again qualitatively sow that their experimental data can distinguish between benign and pathogenic MLH1 variants with some curious exceptions.

The most significant contribution of this manuscript is the generation of comprehensive datasets that provide mechanistic insights into the impact of missense mutations on MLH1.

This provides a valuable resource for understanding MLH1 function and dysfunction. The impact of interface mutations on protein stability also provide an observation that is worth noting as a potential more general trend for future studies with a larger set of protein interactions. This work is generally well-executed and primarily lacks some additional quantifications for some of the observations made in many sections of the work.

Our response:
We thank the reviewer for their overall positive assessment and for taking time to evaluate our work and provide valuable feedback.

1 - Quantification of Relationships: The manuscript makes many qualitative statements or observations that require quantitative support. None of these quantifications are themselves a major concern but overall the manuscript is missing too many of these. Below is a list of examples that I think would benefit from quantification and statistics:

1.1 - AlphaFold2-Multimer iPTM score for the predicted structure and the similarity with the yeast experimental structured.

Our response:
We thank the reviewer for highlighting this shortcoming. The ipTM question overlaps with question 1 from reviewer 1, and we refer to the more detailed answer on pages 1-2 as answering this became more complex than expected. Sequence and structural similarity to the yeast complex are 30% for the MLH1 domain, 42% for the PMS2 domain, and 2.08Å for the structure, which are now reported on line 107-111 of the manuscript.

1.2 – The iPTM score for AlphaFold2-Multimer of the PMS2 α-helix interacting with the central α-helix in MLH1

As discussed on page 1, we have upon closer inspection discarded the model of the 605-627 helix in isolation, as it turned out to not be compatible with the full-length interaction.

1.3 – line 202 - "median abundance scores correlated with relative solvent accessible surface area (rSASA)" add correlation coefficient and p-value

Our response:
We thank the reviewer for pointing this out. We have now added Spearman's correlation coefficients to both plots (new **Supplementary Fig. 7**).

1.4 – line 224 "we performed low-throughput Y2H growth assays" – please describe the results of these experiments in the main text

Our response:
We now describe that these experiments confirmed the effects observed in the high-throughput screen (lines 231-232, in tracked manuscript).

1.5 – lines 268-275 :"We mapped the experimental abundance scores to the predictions, which revealed that variants with wild-type-like abundance clustered in the upper left corner, whereas low-abundant MLH1 variants mapped towards the bottom right corner". Please quantify this – what is the correlation between GEMME, predicted ddG and abundance change, similar for the interaction change in the continuation of the text. One might expect that ddG is better predictor of abundance tan GEMME. Is tis true ?

Our response:
We thank the reviewer for suggesting these analyses. We now include scatter plots correlating Rosetta/GEMME with our abundance/interaction scores (new **Supplementary Fig. 11** and discussion in line 277 in the manuscript). Interestingly, Rosetta and GEMME predict abundance equally well, likely indicating that most of the signal captured by GEMME is related to effects on stability or abundance. This is in line with our observation of most non-wildtype-like variants affecting abundance, but not the interaction.

2 - Predictive Power for Pathogenic Variation: While the manuscript show that the data can qualitatively distinguish between benign and pathogenic variants, it lacks a detailed analysis of the predictive power of the abundance and interaction scores for pathogenicity. The authors should calculate performance metrics such as sensitivity, specificity, and area under the receiver operating characteristic curve (AUC) to quantify how well their data can predict pathogenic variants. This would provide a clearer picture of the clinical utility of their findings. This concern relates to the first but it is important to make this part particularly clear.

2.1 - Please quantify the relation between the experimental results and the human genetic (ClinVar variants and allele frequencies). Are the experimental results predictive of allele frequency and pathogenicity ?

Our response:
We thank the reviewer for highlighting the need for elaboration here. Indeed, our aim was to assess qualitatively how well our experimental data could distinguish between pathogenic and benign variants. Since our data is not intended to be directly used in any clinical decision making, we initially decided not to quantify the predictive power of the generated datasets. However, as suggested by the reviewer, we now include ROC curves for how well our abundance and interaction scores, as well as Rosetta and GEMME predict MLH1 pathogenicity (new **Supplementary Fig. 13**). We describe these results in the revised manuscript (lines 306-

313, in tracked manuscript). We chose not to quantify allele frequency predictions, as this is not a direct metric of pathogenicity – while pathogenic variants are often rare, rare variants are not necessarily pathogenic, in particular given the bias between populations studied (Cassa *et al., Hum Mutat* 2014).

2.1 - Are the prediction models (from Rosetta and GEMME) better or worse in terms of predicting allele frequency and pathogenicity ?

Our response:
As described in the response above, we now include these ROC curves (new **Supplementary Fig. 13**). GEMME serves as the best overall predictor of MLH1 pathogenicity, while Rosetta performs slightly worse than our experimental scores. This has been observed in other studies and is essentially expected, as GEMME assesses the MSA and hence potentially captures all changes that are detrimental to MLH1 function, rather than just those due to stability or abundance. However, importantly, GEMME does not provide mechanistic insights into variant effects, which may make it interesting to use it alongside a more specific predictor or experimental screen.

2.2 - Can the experimental results (stability and interaction) be combined to improve the prediction of the human genetics data ?

Our response:
Indeed, it is possible that combining our abundance and interaction scores would yield more accurate predictions. However, integrating these scores is not straightforward, as even the simplest combination approach will require determining weights for each of the individual measurements. In this work, our aim was to disentangle the mutational effects on abundance and interaction to provide mechanistic insights, rather than probing global MLH1 function. For a specialized MLH1 predictor that integrates multiple sources of data, it may be advisable to also include other information, such as splice sites, transcription, localization etc.

2.3 – Can the experimental results be combined with the prediction models to predict the human genetics data ?

Our response:
This is an interesting and complex question. The typical use of Rosetta stability predictions is as a proxy of stability or abundance data, in absence of access to such experiments. Thus, it would be unusual to use the experimental abundance data together with the predictions, except for tasks where the aim is to improve the predictions.
The situation with the interaction data is slightly different, because neither of the predictors we used is specific for impact on interactions. If available on larger scales and for mutations across multiple different proteins, it could for example be used to learn which positions or mutations

are most likely to affect interactions, to what extent they overlap with those sensitive to abundance and whether they are all identified by general functional models like GEMME. We do not think that the number of mutations we have in this specific study is suitable for such a model though.

line 200: "Multimer-predicted structure of the human C-terminal MutLα complex (Fig. 3B). This revealed that most of the sensitive positions were pointing inwards" – it was not clear to me what "inwards" means here

Our response:
Thank you for pointing out that this was unclear. We now write "into the core of the protein" instead (line 204, in tracked manuscript).

lines 215-221 – There are several structural observations that are made here and I was intrigued by them but it would have been nice to have some ideas by the authors about what might be the underlying structural mechanisms for these examples.

Our response:
In the original manuscript, several of these observations and possible interpretations were placed in the Discussion section (lines 323–336). In the revised manuscript, we now also elaborate on the potential gain-of-interaction mechanism for Q544W (lines 345–349, in tracked manuscript).

In regards to the relation between Rosetta ddG and protein abundance it would be interesting to discuss where there might be some discrepancy between predicted loss of stability and loss of protein abundance. In other words, are there meaningful numbers of mutations that are predicted to be destabilizing but do not result in lower abundance ? Are these mostly potential issues with the Rosetta predictions ?

Our response:
Indeed, a few variants (< 1%) were predicted to be destabilizing by Rosetta but displayed wild-type-like abundance levels (see new **Supplementary Fig. 11A**). We examined these variants, which do not cluster in specific positions, but found enrichment of substitutions to proline or small-to-large substitutions, which are notoriously difficult for Rosetta to predict.
Further, it is also possible for a prediction to be biophysically correct (if the assessment were done *in vitro*) but the cell's protein quality control system (e.g. protein refolding by chaperones) may influence the cellular abundance of the variants.

It is very strange that there are mutations that decrease stability without impact on stability. The most likely scenario is that the Y2H is mostly acting as a qualitative measurement of

interaction and a relatively small amount of protein might be enough to give a positive interaction result. This is mentioned in the discussion and it is very important aspect that may limit some of the conclusions of the work. This is framed in the discussion as the Y2H being more sensitive which was not very obvious and clear to me. Perhaps this part needs to be revised a bit more for clarity.

Our response:
As the reviewer correctly points out, we address this in the Discussion section (lines 337–352) and supplementary material (see also new **Supplementary Fig. 15**), offering several possible explanations for why MLH1 variants may appear unstable yet bind PMS2 similarly to the wild-type in our experimental assays. We now mention this as a key limitation of our work (line 355, in tracked manuscript).

Reviewer #3 Larsen-Ledet and Stein used a yeast screening system to address two molecular effects of practically all possible missense substitutions of the MLH1-C-terminal domain. This domain confers the constitutive dimerization with its heterodimeric partner PMS2. They assessed the consequences of variants using two biochemical readouts: protein stability (DHFR-PCA) and dimerization (yeast two-hybrid).
They were able to confirm certain aspects that may be expected based on traditional knowledge in protein biochemistry: for example, that more detrimental effects of substitutions are conferred in internal residues and in residues located in secondary structures (e.g. PMID 33106425).
The strength of the work is the large number of variants analyzed in this screening which is of interest for those searching for ways to unveil the functional roles of specific residues or the potential of a given human variant to inactivate the protein and thereby be causative for Lynch syndrome. This may facilitate Lynch syndrome diagnosis of uncertain variants (VUS). A general caveat in the interpretation, however, is that “only” two aspects of potential dysfunctionality/pathogenicity (stability and dimerization) have been addressed (and not catalytic MMR activity, for example) and therefore variant neutrality cannot be assumed solely based on negative findings in the assays performed.
Some issues, therefore, need be addressed to render the manuscript complete and accurate for publication.

Our response:
We thank the reviewer for the comments and for taking time to evaluate our work and provide valuable feedback.

Major comment #1
Abstract and throughout the manuscript.

The claim that a yeast-based functional assay provides clinically applicable decisions ("benign" and "pathogenic") goes too far considering that not all possible mechanisms of pathogenicity are being investigated, and that a non-human experimental system is being used (see also https://www.insight-group.org/criteria/ for the procedures used to turn experimental data in clinically applicable decisions). Moreover, the MMR activity, which provides the most complete read-out of the biochemical functionality of a variant MLH1 protein, is not included in this work, neither the endonuclease activity, which is encoded in the MLH1-PMS2 CTD (this activity should also be reported in line 255 of the manuscript and not omitted).
Therefore, the claim to measure whether a variant is neutral or pathogenic must be dropped, it can only be stated if the variant is "functional" or "non-functional" in terms of stability and dimerization.

Our response:
We thank the reviewer for this comment. We regret not being clear in the manuscript that the purpose of this work was not to assess the global functionality of MLH1 variants or provide clinically applicable decisions regarding pathogenicity. Instead, our aim was to explore the mechanistic effects of mutations on MLH1 abundance and its interaction with PMS2, which cannot be achieved using an MMR activity assay. We agree that it is important to highlight that wild-type-like variants observed in our assays should not be presumed to be functional or benign. We now mention this in the revised manuscript (lines 386-389, in tracked manuscript).

Major comment #2
Lines 132ff. Sensitivity and dynamic range were confirmed with variants supposedly neutral. However, for Q542L, which is a VUS, conflicting results have been published, including those that suggest an MMR defect (e.g. PMID 20533529, discussed in PMID 36054288), an information that should be included in the discussion.
Moreover, the authors state correctly that variants in the N-terminal domain cannot be expected to directly impact interaction (lines 154 ff.) However, they detected a complete loss of interaction for the T117M variant located in the N-terminal domain (Fig. 2A, lines 146 ff.). Therefore, it must be concluded that Y2H assay detects loss of interaction as well as loss of stability. Consequently, a variant that is deficient in both assays is not really a bona fide dimerization-deficient variant, only those which are normal in DHFR and deficient in Y2H. This should be clearly stated and discussed appropriately.

Our response:
We have now added this information and citated the suggested papers regarding the Q542L variant in the Results section in the revised manuscript (lines 141-142, in tracked manuscript). The reviewer is correctly pointing out that the Y2H assay cannot discriminate between variants that lose the interaction due to reduced abundance levels and those that are so-called bona fide dimerization-deficient variants. Therefore, we complement the Y2H assay with the DHFR-PCA to measure the cellular abundance of MLH1 variants. We also refer to the Introduction section (lines 67-70) and the Results section (lines 123-126 and lines 145-146) for related discussions.

Major comment #3
Considering the large number of variants tested, it is hardly possible discuss the results with all previous data, but for such work that has explicitly concentrated on human MLH1 stability (PMID 23403630 and 31697235) and dimerization with PMS2 (PMID 20533529) should still be used for comparing the results of the screening to previous measurements, both as a means of verification of the methods and summarizing the current knowledge. Therefore, a some kind of comparison in form of a table or otherwise would add a lot of worthwhile information to the work.

Our response:
We thank the reviewer this suggestion and have now include plots with comparisons (see new **Supplementary Fig. 17**). Overall, we were pleased to see that the measurements reported in the literature are in line with our own.

Minor comments
Line 319 ff.
It may be of interest to add the information that SLiMs have also been identified in MLH1, therefore they too constitute a functionality relevant feature in MLH1-PMS2 biochemistry (PMID 36215471, 32731489 and 37224528).

Our response:
Indeed, MLH1 has been shown to contain SLiMs. However, to our knowledge these motifs are found in the intrinsically disordered linker between the N- and C-terminal domains, and have been shown to be involved in endonuclease activity and nuclear translocation. It is well possible that the putative helices in PMS2 AlphaFold predicts to bind to MLH1 contain SLiMs. In particular, this is an interesting opportunity for follow-up for the more N-terminal helix, which is predicted to bind to the region of MLH1 where we observe changes in binding upon mutation. The new supplementary figure S10 has several residues highlighted. Given that we are working with a single instance here though, we have not attempted to derive a SLiM-type pattern.
