# Supplementary material for: Disentangling the mutational effects on protein stability and interaction of human MLH1
Source: PLoS Genet. 2025 Apr 28;21(4):e1011681. doi: 10.1371/journal.pgen.1011681 (PMC12064032; doi:10.1371/journal.pgen.1011681)
Supplement: Attachment — Submitted filename: Response to Reviewers_Re-revision_v2.docx [file pgen.1011681.s021.docx]

**Our point-by-point response to the raised issues**

**Reviewer #1** The authors have addressed my concerns. Please put the unit (Angstrom) for the RMSD reported on p.6.

Our response:

We thank the reviewer for taking the time to evaluate our work and for facilitating an efficient revision process. We now include units for the RMSD in the revised manuscript (line 113).

**Reviewer #2** The authors have address all major concerns that I had previously.

Our response:

We thank the reviewer for taking the time to evaluate our work and for facilitating an efficient revision process.

**Reviewer #3** Overall, the authors have adequately incorporated the suggested improvements.

Our response:

We thank the reviewer for taking the time to evaluate our work and for facilitating an efficient revision process.

Concerning the previous “major point 1”, though the authors have correctly addressed this issue, however, the sentence in the abstract “Our data successfully DISTINGUISH benign from pathogenic MLH1 variants…”, referring to Figure 4, still is rather misunderstandingly suggesting that clinical decisions are a capacity of the applied assays. Please change to make clear that the acquired data CORRELATED with clinical classifications of the investigated variants (as shown in Figure 4), to make sure that this point is clear.

Our response:

We have now changed the wording in the abstract as suggested in the revised manuscript (line 29).

Minor comment:

Figure 4: In the Figure legend, it is stated that variants without gnomAD occurrence are “indicated to the left of the dashed lines”. There is no dashed line, rather the “axis break” in the x-axis is meant here. Please either introduce a dashed line or correct in the legend.

Our response:

We thank the reviewer for pointing this out. We have now changed the wording in the figure legend as suggested in the revised manuscript (line 1067).
